# Collision tumor of endometrial hepatoid adenocarcinoma and endometrial stromal sarcoma: a rare case and literature review

**DOI:** 10.3389/fonc.2025.1661354

**Published:** 2026-01-16

**Authors:** Jie Zhang, Dengfeng Wang, Min Shi, Ting Zhou, Xunwei Shi, Liping Peng, Yang Liu, Guonan Zhang

**Affiliations:** 1 Department of Gynecologic Oncology, Sichuan Provincial Key Medical Laboratory of Gynecologic Oncology, Health Commission of Sichuan Province, Sichuan Clinical Research Center for Cancer, Sichuan Cancer Hospital & Institute, Sichuan Cancer Center, University of Electronic Science and Technology of China, Chengdu, China; 2 Department of Pathology, Sichuan Clinical Research Center for Cancer, Sichuan Cancer Hospital & Institute, Sichuan Cancer Center, University of Electronic Science and Technology of China, Chengdu, China; 3 Department of Radiology, Sichuan Clinical Research Center for Cancer, Sichuan Cancer Hospital & Institute, Sichuan Cancer Center, University of Electronic Science and Technology of China, Chengdu, China

**Keywords:** endometrial hepatoid adenocarcinoma, endometrial stromal sarcoma, collision tumor, chemotherapy, oncology, PET/CT (^18^F-FDG)

## Abstract

**Background:**

Endometrial hepatoid adenocarcinoma (HAC) is an exceedingly rare tumor. Histologically, the tumor is predominantly characterized by HAC with additional components, as documented in previous reports. The coexistence of endometrial HAC and endometrial stromal sarcoma in collision tumors is exceptionally uncommon. To our knowledge, this case represents only the second documented instance of such a collision tumor.

**Case presentation:**

A 69-year-old postmenopausal woman presented with abnormal uterine bleeding, and her serum alpha‐fetoprotein (AFP) concentration was 8926 ng/mL. The initial biopsy revealed poorly differentiated adenocarcinoma. Subsequent postoperative histopathological evaluation confirmed the coexistence of endometrial HAC and endometrial stromal sarcoma. Notably, according to imaging, gross examination, and microscopy, the two tumor components were separated by distinct contact areas without evidence of migration or intermingling. The patient developed pulmonary metastases within 40 days of surgery and eventually died of the disease.

**Conclusion:**

Endometrial hepatoid adenocarcinoma is an exceptionally rare tumor that frequently coexists with other pathological components. Preoperative diagnosis remains challenging, and definitive characterization relies predominantly on postoperative evaluation, particularly immunohistochemical analysis. These tumors exhibit marked heterogeneity and aggressiveness, with a propensity for lung metastasis and an overall poor prognosis. Due to the limited number of reported cases, there is no established consensus regarding the optimal treatment. Some case reports suggest that the development of hepatoid adenocarcinoma of the uterus may have some correlation with endometriosis. ^18^F-Fluorodeoxyglucose (^18^F-FDG) positron emission tomography/computed tomography (PET/CT) enables the comprehensive pretreatment assessment of disease extent and the early detection of metastases. Surgical intervention remains the mainstay of treatment, but accurate evaluation of pathological characteristics is critical for selecting the optimal chemotherapy regimen. In this context, the CAP regimen—comprising cyclophosphamide, adriamycin, and cisplatin—has demonstrated superior overall efficacy. Moreover, these tumors seem to be relatively resistant to radiation therapy.

## Background

AFP-producing tumors in the uterus include malignant mixed mesodermal tumors (MMMTs), hepatoid carcinomas, and yolk sac tumors. We present a case of AFP-producing endometrial hepatoid adenocarcinoma that formed a collision tumor with endometrial stromal sarcoma, an exceptionally rare phenomenon. This case represents only the second reported instance of a collision tumor involving endometrial HAC. Additionally, we reviewed case reports of endometrial HAC and summarized its clinicopathological characteristics.

## Case presentation

**Figure 1:**
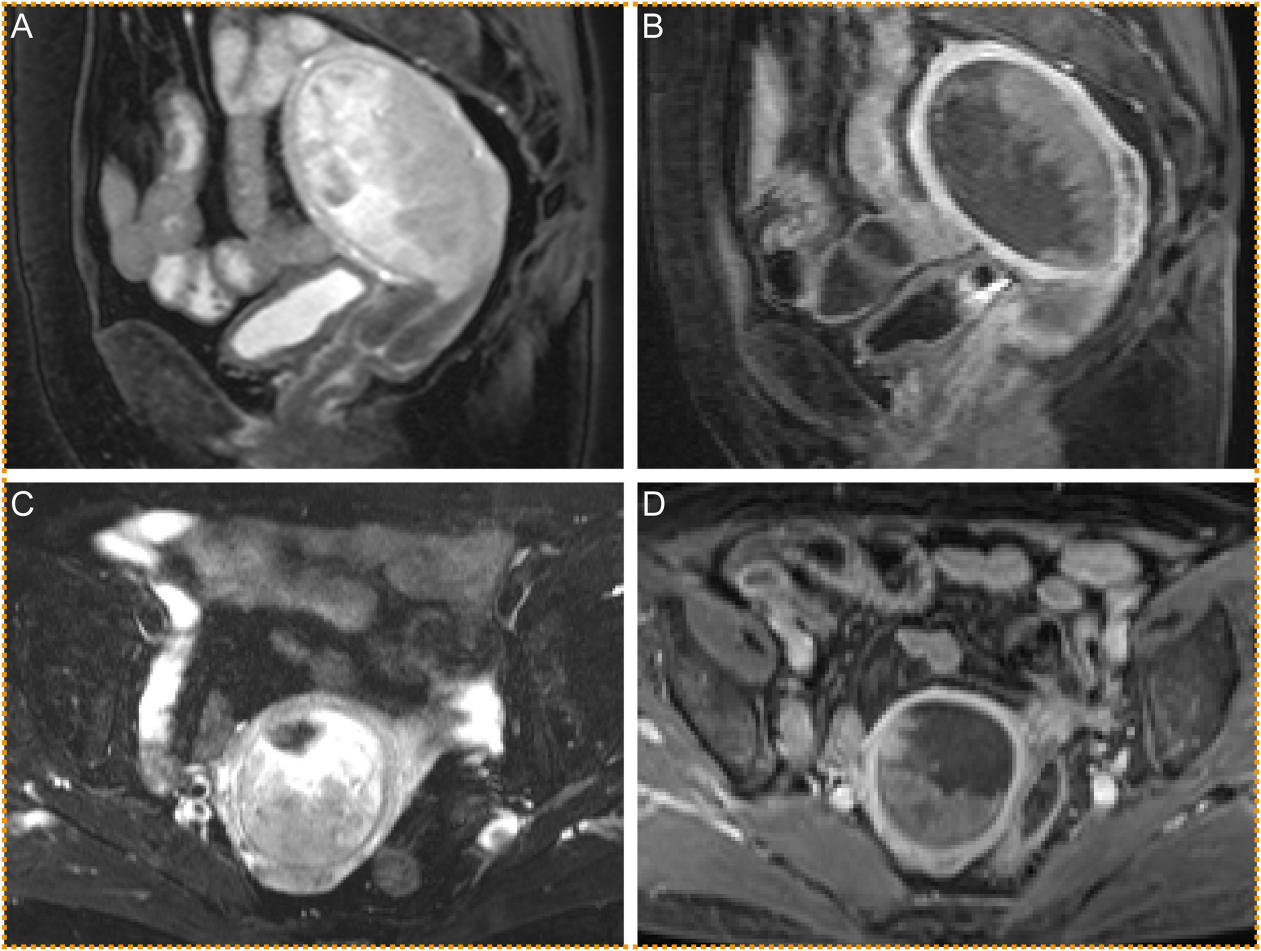
Sagittal T2 **(A)** and enhanced sagittal **(B)** sequences show two signals in the uterine cavity of a tumor colliding at a clear boundary. Transverse T2 **(C)** and enhanced transverse **(D)** sequences also show a relatively clear boundary between the two signals of the tumor in the uterine cavity.

A 69-year-old female (gravida 2, para 2) presented with a history of vaginal bleeding and lower abdominal pain persisting for two weeks. Menopause was reached at 44 years of age, and she underwent cholecystectomy at age 63 due to gallbladder stones. There was no family history of malignancy and no significant psychosocial history, and the patient did not report weight loss, fever, or other systemic symptoms. Vaginal and pelvic examinations revealed a slightly enlarged, nontender, and smooth uterus, with no palpable adnexal masses or abnormalities in the vaginal wall or portio vaginalis. Laboratory evaluation revealed a markedly elevated serum AFP level of 8926 ng/mL (normal <7 ng/mL), whereas the levels of other tumor markers, such as carcinoembryonic antigen (CEA) and carbohydrate antigen 125 (CA 125), were within normal ranges. Blood counts and serum biochemical profiles were unremarkable. Magnetic resonance imaging (MRI) revealed an enhanced mass measuring 4.9 × 5.4 × 6 cm in the uterine corpus cavity (**Figure 1**), which was highly suggestive of a malignant tumor. No abnormalities were detected in the liver, bile duct, ovaries, pelvic free fluid, or lymph nodes. Computed tomography (CT) revealed micronodules in the posterior segment of the upper lobe of the right lung and in the right horizontal fissure, which were interpreted as inflammatory in nature. On the basis of these clinical, radiological, and laboratory findings, a malignant uterine tumor was strongly suspected. Consequently, dilatation and curettage were performed, and histological examination confirmed the presence of a poorly differentiated malignant tumor. The patient subsequently underwent total abdominal hysterectomy with bilateral salpingo-oophorectomy, pelvic and para-aortic lymphadenectomy, and omentectomy.

**Figure 2:**
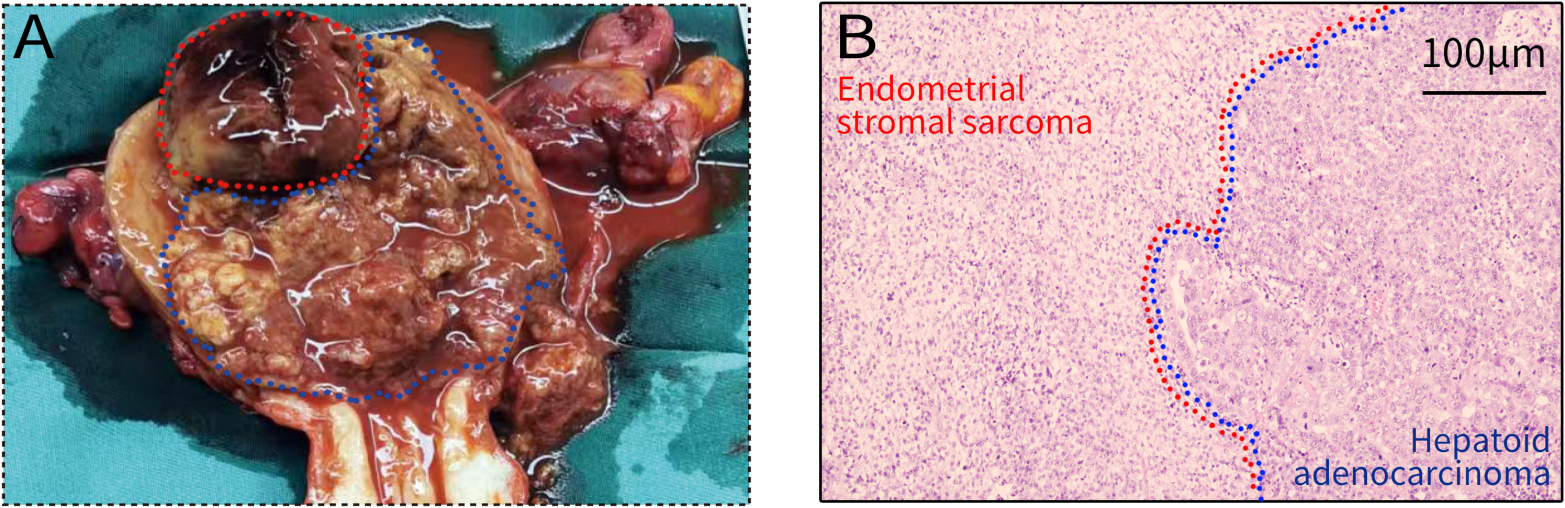
**(A)** The spherical, dark red tumor at the upper part of the lesion exhibits irregular borders and is considered to be endometrial stromal sarcoma. The surrounding necrotic, friable, and congested tumor tissue is interpreted as endometrial hepatoid adenocarcinoma. **(B)** The boundary between hepatoid adenocarcinoma and endometrial stromal sarcoma (Hematoxylin & eosin, Original magnification 100 x). The left area is endometrial stromal sarcoma and the right area is hepatoid adenocarcinoma. The two tumors collide at the borderline.

The resected uterus was slightly enlarged. A dark red exophytic solid polypoid tumor measuring 3.5 × 3 × 4 cm was attached to the endometrium of the posterior part of the uterine fundus. The lower segment of the uterine cavity was filled with necrotic, friable, congested tumor (**Figure 2A**). The two tumor types collided in the uterine body at a clear boundary, infiltrating more than half the thickness of the myometrium and invading the cervical canal. No tumors were identified outside the uterus.

**Figure 3:**
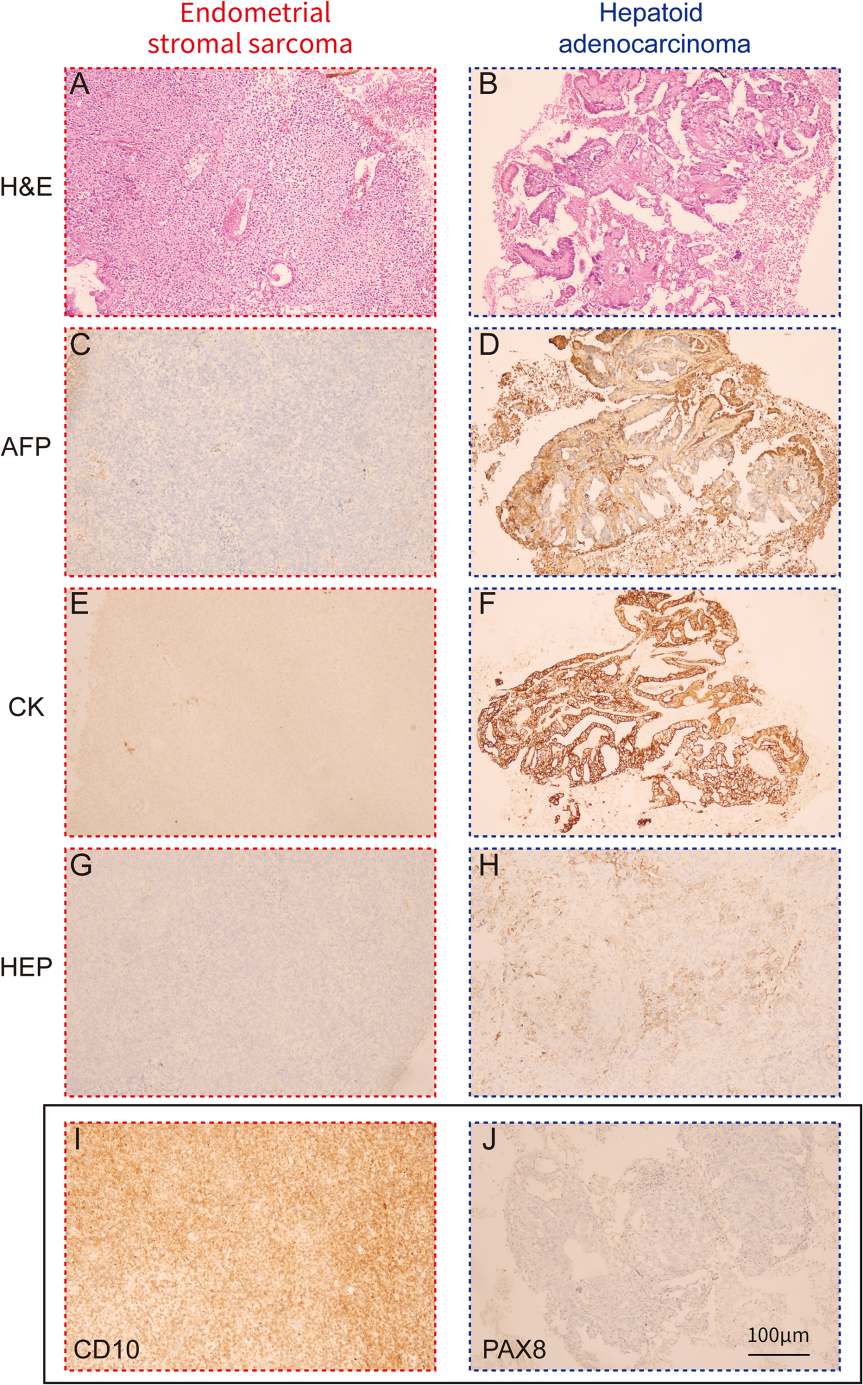
Immunohistochemical analysis results of tumors. **(A)** Photomicrograph of endometrial stromal sarcoma (H & E, 100 x). **(B)** Photomicrograph of hepatoid adenocarcinoma, resembling hepatocellular carcinoma (H & E, 100 x). **(C)** AFP negative in the endometrial stromal sarcoma tumor cells (100 x). **(D)** Immunohistochemical staining for AFP shows positive reactions in the hepatoid adenocarcinoma tumor cells (100 x). **(E)** CK negative in the endometrial stromal sarcoma tumor cells (100 x). **(F)** CK positivity in the hepatoid adenocarcinoma tumor cells (100 x). **(G)** HEP negative in the endometrial stromal sarcoma tumor cells (100 x). **(H)** HEP positivity in the hepatoid adenocarcinoma tumor cells (100 x). **(I)** CD10 positivity in the endometrial stromal sarcoma tumor cells (100 x). **(J)** PAX8 negative in the hepatoid adenocarcinoma tumor cells (100 x).

Microscopic examination revealed two components with distinct morphological features. One component exhibited an adenoid arrangement and was moderately to poorly differentiated, accounting for approximately 80% of the entire tumor. Immunohistochemical analysis demonstrated that tumor cells in this region were positive for AFP, CK, HEP, HNF-1, EMA, CK7, and Cyclin D1, while negative for CD10, PAX8, WT-1, CK8/18, Arg-1, and PTEN (**Table 1**, **Figure 3**), suggestive of adenocarcinoma with hepatoid differentiation. The other component showed a solid sheet-like nest arrangement, comprising approximately 20% of the tumor. Immunohistochemical analysis revealed that tumor cells in this region were positive for Vimentin, CD10, Cyclin D1, and PTEN, while negative for AFP, CK, HNF-1, PAX8, WT-1, EMA, CK7, CK8/18, Arg-1, and HEP (**Table 1**, **Figure 3**), indicating a mesenchymal origin. The two components met at a distinct border within the uterine corpus (**Figure 2B**). Based on these features, the pathological diagnosis was established as “Collision tumor of endometrial hepatoid adenocarcinoma and endometrial stromal sarcoma.” Moreover, the lesion was confined to the uterine corpus, and no adenocarcinoma cells were detected in the peritoneal washing fluid.

**Table 1:** Immunohistochemical marker expression results and their significance in differential diagnosis.

IHC markers	Hepatoid adenocarcinoma	Endometrial stromal sarcoma	The significance of differential diagnosis
AFP	+	–	An embryonic yolk sac protein. Hepatocellular adenocarcinoma may express.
CKp	+	–	Broad-spectrum cytokeratin. Supports epithelial differentiation upon expression
HEP	+	–	Hepatocyte marker. Hepatocellular adenocarcinoma may express.
HNF-1	+	–	Hepatocyte Nuclear Factor-1. Hepatocellular adenocarcinoma may express.
EMA	+	–	Cell surface glycoproteins. Express support for epithelial differentiation.
CK7	+	–	Supports glandular epithelial differentiation upon expression. Hepatocellular adenocarcinoma may express.
Cyclin D1	+	+	Cyclin. High-grade endometrial sarcomas frequently express. Hepatocellular adenocarcinoma occasionally expresses.
CD10	–	+	A Zinc-Dependent Cell Membrane Metal-Binding Protein. Endometrial stromal sarcoma expression. Hepatocellular adenocarcinoma shows minimal expression.
PAX8	–	–	Müllerian-derived epithelial tumors expressing. Sarcomas show minimal expression.
WT-1	–	–	Uterine leiomyosarcoma commonly expresses. But it doesn’t necessarily express.
CK8/18	–	–	Hepatocellular adenocarcinoma may express. But not necessarily expressed.
Arg-1	–	–	Hepatocyte marker. Hepatocellular adenocarcinoma may express.
PTEN	–	+	Liver-like adenocarcinoma may exhibit loss of expression
Vimentin	/	+	Wave protein. Broad-spectrum markers for mesenchymal tumors.

**Figure 4:**
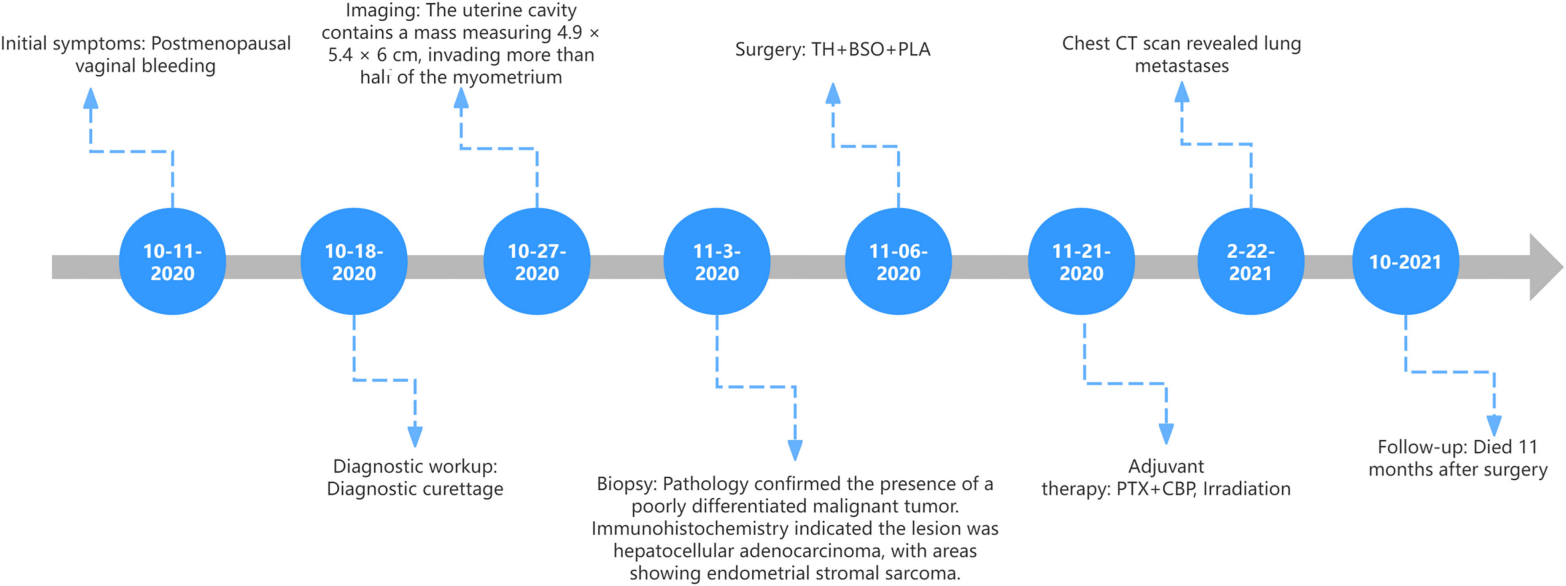
The clinical timeline figure.

The day after surgery, the patient’s serum AFP level decreased to 3950 ng/mL and further decreased to 1519 ng/mL three days later. Two weeks after surgery, a drip infusion regimen of 175 mg/m² paclitaxel combined with carboplatin (AUC = 5) was initiated every three weeks. One month after surgery, the patient commenced external beam radiotherapy to the pelvic region on December 4, 2020, at a dose of 1.8 Gy/28 fractions. From February 3 to February 5, 2021, the patient completed two sessions of 3D-IGRT, achieving a HRCTV of 600 cGy and an IRCTV of 500 cGy. Although her serum AFP level decreased to 15.82 ng/mL, normalization was not achieved before the initiation of her second chemotherapy cycle. Three months postoperatively, a computed tomography scan of the chest revealed multiple nodules in the upper lobes of both lungs, which had increased in number and size compared with previous scans, with the largest nodule in the right lung’s lower lobe measuring approximately 2.5 × 1.7 cm, indicative of lung metastasis. At that time, her serum AFP level had increased to 121.20 ng/mL. It is recommended to switch to an alternative chemotherapy regimen combining CPA, ADM, and CDDP with targeted therapy. However, considering the patient’s advanced age and poor tolerance to side effects from previous dual-drug chemotherapy, coupled with refusal of targeted therapy due to lack of insurance coverage, the patient and family ultimately opted for single-agent cisplatin chemotherapy. The third cycle chemotherapy regimen was adjusted to daily intravenous cisplatin 30mg on days 1-3. Unfortunately, the pulmonary metastases continued to progress, with the largest lesion enlarging to 3.8 × 2.5 cm. Due to recurrent chemotherapy-induced bone marrow suppression, the patient was discharged on March 31, 2021. Follow-up revealed the patient received two additional chemotherapy cycles at a local hospital. Ultimately, more than 11 months after surgery, the patient passed away due to complications from the pulmonary metastases (**Figure 4**).

## Discussion

HAC is a variant of adenocarcinoma characterized by hepatic differentiation. It belongs to a heterogeneous group of tumors that are morphologically and immunophenotypically similar to hepatocellular carcinoma but that originate from outside the liver. The concept of HAC, an AFP-producing cancer exhibiting hepatic differentiation that originates in the stomach, was first introduced in 1985 (1). HAC has been identified in several organs, including the stomach, lungs, mediastinum, gallbladder, pancreas, bladder, ovaries, rectum, renal pelvis, vagina, and uterus, with the stomach being the most common site (2). Although the number of case reports of AFP-producing adenocarcinomas aside from hepatocellular carcinoma has increased, AFP-producing adenocarcinomas of the endometrium remain exceedingly rare. This report describes a case in which the patient presented with a collision tumor comprising endometrial HAC and endometrial stromal sarcoma. Typically, a collision tumor is defined as the coexistence of two or more histologically distinct tumors within the same organ without evidence of histologic intermixing. Most documented collision tumors occurred in the brain, skin and urological system. In this instance, the two tumors in the endometrium remained clearly distinct, as confirmed by both gross and microscopic examination. To date, only one other case involving an endometrial HAC collision tumor has been documented (3), making this case the second such case.

We reviewed reported cases of primary endometrial HAC and identified 15 case reports (**Table 2**) comprising 16 patients. These patients were predominantly older postmenopausal women, with a mean age of 64 years. Notably, all patients presented elevated preoperative serum AFP levels, ranging from 117 to 110,610 ng/mL. The follow‐up data revealed that nine patients remained disease‐free for periods ranging from 8 months to 8 years, although follow‐up information was unavailable for three patients, complicating the estimation of progression‐free survival. Previously, the one‐year survival rate for HAC was reported to be 55%, with a median overall survival of 11 months (2). Among the 16 patients, five died from the disease, with one case lacking a documented cause of death. Notably, four of the five deceased patients developed lung metastases (4–8), mirroring the clinical course observed in the current report. In our patient, pulmonary metastasis was detected more than 40 days post‐surgery, and the disease progressed rapidly; despite intensive modifications to the chemotherapy regimen, the lung metastases worsened, and the patient died approximately 11 months after surgery. These findings underscore the pronounced susceptibility to pulmonary metastasis as a characteristic feature of endometrial HAC.

**Table 2:** Summary of 16 cases of endometrial hepatoid adenocarcinoma.

Case	Authors	Year	Age (yrs)	Symptoms	AFP (ng/mL)	Surgery	Adjuvant therapy	Histology	Stage	Outcome	Ref
1	Matsukuma	1988	63	Wet cough and sputum	670	TH+BSO	CPA+ADM+CDDP	Poorly differentiated adenocarcinoma	IIB (T1b, N0, M0)	Died 12 months after surgery	(4)
2	Hoshida	1996	66	Postmenopausal vaginal bleeding	16,170	TH+BSO+PLA	Irradiation	Hepatoid adenocarcinoma, endometrioid adenocarcinoma	IIIC (T2b, N1, M0)	Died 32 months after surgery	(10)
3	Yamamoto	1996	62	Postmenopausal vaginal bleeding	280.3	TH+BSO	CPA+ADM+CDDP	Endometrioid adenocarcinoma, Hepatoid adenocarcinoma	IVB (T1c, Nx, M1)	Died 4 months after surgery	(6)
4	Hongyue Sun	1997	63	Postmenopausal vaginal bleeding	ND	TH+BSO	Irradiation Chemotherapy without details	Endometrioid adenocarcinoma, Hepatoid adenocarcinoma	IA (T1a, N0, M0)	Alive without disease 14 months after surgery	(11)
5	Toyoda	2000	60	Postmenopausal vaginal bleeding	31,950	TH+BSO+PLA	CPA+ADM+CDDP	Endometrioid adenocarcinoma, Hepatoid adenocarcinoma	IIIC (T1b, N1, M0)	Died 12 months after surgery	(7)
6	Adams	2001	66	Postmenopausal vaginal bleeding	351	TH+BSO+PLA	CPA+ADM+CDDP	Hepatoid adenocarcinoma, endometrioid adenocarcinoma	IIIA (T3a, N0, M0)	Alive without disease 8 years after surgery	(12)
7	Takano	2003	63	Postmenopausal vaginal bleeding	5060	TH+BSO+PLA	PTX+CBP	Endometrioid adenocarcinoma, hepatoid, smooth muscle sarcoma and rhabdomyosarcoma	IIIA (T3a, N0, M0)	Short FU: 12 months	(20)
8	Takahashi	2003	68	Postmenopausal vaginal bleeding	2800	TH+BSO	ND	Collision tumor (Hepatoid adenocarcinoma, carcinosarcoma)	IIB (T2b, N0, M0)	ND	(3)
9	Takeuchi	2006	61	epigastric discomfort	453	TH+BSO+PLA+omentectomy	PTX+CBP	Hepatoid adenocarcinoma, endometrioid adenocarcinoma	IVB (T1b, N0, M1)	Short FU: 12 months	(13)
10	Zhi sun	2008	56	Postmenopausal vaginal bleeding	>3000	TH+BSO+PLA	CPA+ADM+CDDP Irradiation	Endometrioid adenocarcinoma, Hepatoid adenocarcinoma	IA (T1a, N0, M0)	Alive without disease 30 months after surgery	(14)
11	Jianbo Wu	2009	46	Postmenopausal vaginal bleeding	117	TH+BSO+PLA	ND	Endometrioid adenocarcinoma, Hepatoid adenocarcinoma	IA (T1a, N0, M0)	Short FU: 8 months	(15)
12	Kawaguchi	2011	63	Postmenopausal vaginal bleeding	10,131	TH+BSO+PLA	PTX+CBP	Hepatoid adenocarcinoma, Rhabdomyosarcoma	II (T2, N0, M0)	Alive without disease 24 months after surgery	(8)
13			82	Postmenopausal vaginal bleeding	401	TH+BSO	PTX+CBP	Endometrioid adenocarcinoma, Hepatoid adenocarcinoma, rhabdomyosarcoma	IB (T1b, N0, M0)	DOD: 1 year	(8)
14	Ishibashi	2011	86	Postmenopausal vaginal bleeding	7824	TH+BSO	Irradiation Oral administration of VP-16	Endometrioid adenocarcinoma, Hepatoid adenocarcinoma	I A (T1a, N0, M0)	Alive with swollen pelvic lymph nodes 36 months after surgery	(16)
15	Xiangyu Cai	2020	63	Postmenopausal vaginal bleeding	543.91	TH+BSO+PLA	PTX+CBP	Endometrioid adenocarcinoma, Hepatoid adenocarcinoma	IB (T1b, N0. M0)	ND	(17)
16	Dejanovic	2022	57	Postmenopausal vaginal bleeding	110,610	No surgery	PTX+CBP Sorafenib	Endometrioid adenocarcinoma, Hepatoid adenocarcinoma	IVB (Tx, Nx, M1)	ND	(18)

ND, not described; TH, total hysterectomy; BSO, bilateral snlpingo-oophorectomy; PLA, pelvic lymphadenectomy; CDDP, cis-platinum; ADM, adriamycin; CPA, cyclophosphamide; VCR, Vincristine; FU, follow-up; PTX, paclitaxel; CBP, carboplatin; VP-16, Etoposide.

Endometrial hepatoid adenocarcinoma frequently exhibits exophytic growth within the uterine cavity. Histologically, it is generally a high-grade malignancy that proliferates in solid, lamellar, trabecular, or cord-like arrangements. The pathological diagnosis of HAC from small biopsy samples prior to surgery is challenging and typically relies on postoperative analyses, especially immunohistochemistry, which reveals AFP expression in HAC cells. Owing to its rarity, specific management guidelines for HAC are lacking; consequently, treatment generally follows the principles established for endometrial cancer. Surgical intervention is typically recommended as the primary modality and is often supplemented by adjuvant chemotherapy and/or radiation. However, given its considerable heterogeneity, accurate pathological identification is essential for selecting appropriate chemotherapeutic regimens. A review of the literature indicates that intrauterine AFP-producing tumors may include malignant mixed Müllerian tumors (MMMTs), hepatoid carcinomas, yolk sac tumors, and occasionally, plasmacytoid carcinoma of the uterus in the absence of hepatoid cells (5, 9).

A feature distinguishing endometrial hepatoid adenocarcinoma from other AFP-producing tumors of the uterine corpus is the presence or absence of hepatoid cellular differentiation on cytomorphological examination, with all tumor cells typically exhibiting immunohistochemical positivity for AFP. Endometrial HAC is frequently associated with additional pathological components, most commonly endometrial adenocarcinoma (6, 7, 10–18) and, in some cases, a sarcomatous component (3, 8, 19, 20). Consequently, the heterogeneity of pathological constituents necessitates that chemotherapy protocols be specifically tailored to address the unique composition of each case. CAP or TC regimens (combining paclitaxel and carboplatin) are commonly used as chemotherapeutic strategies according to recent literature, and treatment involving sorafenib has also reported following disease progression.

The patient described in this case was diagnosed with a collision tumor—a rare tumor, especially since it occurred in the uterus. The differential diagnosis should also include carcinosarcoma and yolk sac tumor. However, considering the patient’s age, tumor location, morphological characteristics, and immunophenotype, we believe the diagnosis of collision tumor of endometrial hepatoid adenocarcinoma and endometrial stromal sarcoma is most appropriate. First, regarding carcinosarcoma, the 5th edition of the WHO Classification of Female Genital Tumors clearly categorizes uterine carcinosarcoma under endometrial carcinoma, specifically as carcinoma of Müllerian origin, which commonly expresses PAX8. However, the epithelial component in this case was PAX8-negative. The sarcomatous region in this case showed positivity for CD10 and Vimentin, with absence of ER/PR, BCOR, and molecular testing results (JAZF1, YWHAE, BCOR rearrangement), providing very limited information for further classification. Nevertheless, considering the specific location within the uterine corpus, endometrial stromal sarcoma remains statistically more common. Therefore, although some diagnostic uncertainty persists, our diagnosis represents the most optimal consideration. Second, when hepatoid differentiated tumors occur in the female reproductive system, distinction from yolk sac tumor is required. However, yolk sac tumor is rare in elderly women and uncommon in the uterus. Moreover, a diagnosis of yolk sac tumor cannot account for the presence of the sarcomatous component. Additionally, it is somewhat regrettable that Arg-1 was negative in the epithelial component of this case. However, as a more specific hepatocellular marker, the limitation of Arg-1 lies precisely in its poor sensitivity; thus, a negative Arg-1 result does not exclude hepatoid adenocarcinoma.

This report documents for the second time the phenomenon of endometrial HAC colliding with other pathological components to form a collision tumor, an extremely rare pathological manifestation. Currently, no standard treatment guidelines exist for uterine collision tumors; hence, a comprehensive approach involving surgery, radiotherapy, and chemotherapy is recommended. Given the highly aggressive nature of endometrial HAC, we administered the TC chemotherapy regimen post-surgery, which resulted in a significant decrease in the patient’s AFP levels. Although radiotherapy was also employed, it did not appear to increase survival, and in contrast, its side effects—including grade IV myelosuppression and thrombocytopenia—compromised the chemotherapy regimen. Furthermore, the development of lung metastases 41 days after surgery suggests the possibility that microscopic lung metastases were present at the time of diagnosis but undetectable on initial imaging.

Preoperative whole-body ^18^F-FDG PET/CT is recommended for patients with endometrial HAC because of the elevated risk of early distant metastases, particularly to the lungs. Multiple reports have documented fatalities resulting from lung metastases in similar cases (4, 6–8), underscoring the necessity of a comprehensive preoperative evaluation. In this context, ^18^F-FDG PET/CT offers distinct advantages by detecting early metabolic changes, which may precede detectable structural alterations on conventional imaging. For example, Dejanovic et al. (18) reported a case in which ^18^F-FDG PET/CT successfully identified bone, vaginal, and lymph node metastases. Therefore, this modality has significant potential in accurately staging primary endometrial HAC by revealing distant metastases that are not evident on CT alone. Moreover, the possibility of an AFP-producing adenocarcinoma of the endometrium should be considered in postmenopausal women—even in the absence of vaginal bleeding—when conventional imaging fails to confirm the diagnosis. In such cases, ^18^F-FDG PET/CT is invaluable for identifying the primary site of the disease.

During the literature review, several specific types of AFP-producing tumors of the female reproductive system were identified. Tran et al. (9) reported a case in 2007 of a 44-year-old nulliparous woman presenting with a one-year history of abnormal uterine bleeding and pelvic pain. Her preoperative serum AFP level was elevated to 1493 ng/ml. Magnetic resonance imaging revealed an 11.1 × 10 × 7 cm multiseptated cystic mass with increased vascularity located in the pouch of Douglas near the rectosigmoid junction; the endometrium was thickened but did not exhibit significant enhancement. The initial clinical impression was a primary ovarian yolk sac tumor, prompting a comprehensive staging workup for ovarian malignancy. Surgical findings revealed enlargement of both ovaries (right: 11 × 8 × 5 cm; left: 10.5 × 8.5 × 5.5 cm) and the presence of an 8 × 3.5 × 3 cm fleshy, focally necrotic polypoid mass within the uterus, which had invaded approximately 70% of the myometrium and extended into the uterine cervix. Histological examination ultimately revealed that the lesion was a uterine AFP-producing papillary serous carcinoma with metastasis to the ovaries. This case was noted for its unusual clinical and radiological presentation, as well as the relatively young age of the patient.

Kubo et al. (5) reported a case of uterine papillary adenocarcinoma in which immunohistochemical analysis confirmed AFP production by tumor cells despite the histology displaying typical papillary adenocarcinoma characteristics. Similarly, Kodama et al. (21) described a case of AFP-producing endometrial adenocarcinoma in which no histopathological sections exhibited HAC cells, and only partial AFP immunoreactivity was observed in the tumor cells. In both cases, a diagnosis of primary uterine HAC was not made, given the absence of any microscopically identifiable HAC components despite AFP production.

The pathophysiological mechanisms underlying HAC remain elusive. In contrast to previously described cases, Qiu et al. (22) reported an unusual instance of HAC in the female reproductive system. The present case involved a 42-year-old premenopausal woman with a preoperative serum AFP level of 4267.76 ng/mL. MRI revealed an irregular mass on the left posterior side of the uterus, measuring approximately 11 × 6 × 5 cm, with poorly defined margins between the mass and the posterior uterine wall. Notably, no significant abnormalities were observed in the uterus proper or the bilateral adnexa. Microscopic examination revealed endometriosis, and subsequent immunohistochemical analysis confirmed a diagnosis of HAC originating from pelvic endometriosis. This suggests that the development of endometrial hepatoid adenocarcinoma may be associated with endometriosis. As additional cases are reported, these findings may provide further insights into the pathophysiological mechanisms underlying endometrial HAC.

## Conclusion

Endometrial hepatoid adenocarcinoma is an exceedingly rare malignancy, and collision tumors in this context are even less common. In the absence of standardized treatment protocols, diagnostic approaches are derived from established principles for endometrial cancer. Some case reports have suggested a potential association between the development of hepatoid adenocarcinoma of the uterus and endometriosis. A review of the literature revealed that endometrial HAC has a high propensity for early pulmonary metastasis. Comprehensive evaluation using ^18^FDG-PET/CT facilitates the detection of metabolic alterations that precede structural changes, thereby enabling early identification of metastases and informing the development of effective therapeutic strategies. Precise pathologic characterization is crucial for selecting the most appropriate chemotherapy regimen. In addition to surgical intervention, chemotherapy plays a critical role in treatment. The reported chemotherapeutic regimens primarily included CAP or TC, with evidence indicating that compared to traditional regimens, CAP may exhibit increased efficacy and prolong survival in some patients. In contrast, radiotherapy appears to be less effective, particularly in patients with a sarcomatous component, owing to the limited radiosensitivity of this component and the potential for aggressive regimens to exacerbate treatment-related side effects, which may delay the completion of chemotherapy and ultimately compromise overall outcomes.

## Data Availability

The original contributions presented in the study are included in the article/supplementary material. Further inquiries can be directed to the corresponding author.
